# Sex Differences in the Expression of Drug-Metabolizing and Transporter Genes in Human Liver

**DOI:** 10.4172/2157-7609.1000119

**Published:** 2012-07-10

**Authors:** Lun Yang, Yan Li, Huixiao Hong, Ching-Wei Chang, Li-Wu Guo, Beverly Lyn-Cook, Leming Shi, Baitang Ning

**Affiliations:** 1 Division of Systems Biology, National Center for Toxicological Research, Food and Drug Administration, Jefferson, Arkansas 72079, USA; 2 Division of Personalized Nutrition and Medicine, National Center for Toxicological Research, Food and Drug Administration, Jefferson, Arkansas 72079, USA; 3 Office of Associate Director of Regulatory Activities, National Center for Toxicological Research, Food and Drug Administration, Jefferson, Arkansas 72079, USA

**Keywords:** Sex difference, Drug metabolizing enzymes, Transporters, Gene expression, Human liver, DMET, Co-expression network analysis

## Abstract

Human sex differences in the gene expression of drug metabolizing enzymes and transporters (DMETs) introduce differences in drug absorption, distribution, metabolism and excretion, possibly affecting drug efficacy and adverse reactions. However, existing studies aimed at identifying dimorphic expression differences of DMET genes are limited by sample size and the number of genes profiled. Focusing on a list of 374 DMET genes, we analyzed a previously published gene expression data set consisting of human male (n=234) and female (n=193) liver samples, and identified 77 genes showing differential expression due to sex. To delineate the biological functionalities and regulatory mechanisms for the differentially expressed DMET genes, we conducted a co-expression network analysis. Moreover, clinical implications of sex differences in the expression of human hepatic DMETs are discussed. This study may contribute to the realization of personalized medicine by better understanding the inter-individual differences between males and females in drug/xenobiotic responses and human disease susceptibilities.

## Introduction

Sex differences in disease susceptibility, drug efficacy, and drug safety have been observed widely in epidemiological studies as well as in clinical reports [1]. In addition, sex differences in the expression of DMETs are thought to be one of the most important determinants accounting for individual differences in clinical pharmacology, pharmacokinetics, and pharmacodynamics [2].

Sex differences in drug metabolism have long been recognized. For example, in 1932, Nicholas and Barron reported that the administration of just one-half of the dosage of sodium amytal needed to anaesthetize male rats could sufficiently anaesthetize female rats [3]. Later, it was found that some drugs were metabolized by certain isoforms of cytochrome P450 with higher rates in male than in female rats (reviewed in [4]). The biochemical basis of sex differences in drug metabolism was also shown to be related to hormonal regulation of the production of drug metabolizing enzymes in animals and humans [5]. During the last several decades, sex differences in drug responses have been extensively investigated using multiple approaches, such as clinical pharmacology, pharmacogenetics, pharmacokinetics, and pharmacodynamics. This effort attempts to provide information to allow a better understanding of the biological basis of sex differences in order to improve public health.

Drug response and efficacy are highly dependent on the bioavailability, distribution, metabolism, and elimination of a drug, all of which are processes driven primary by enzymes. Thus, sex differences in the expression of DMETs play a vital role in determining sex differences in drug efficacy and safety. Sex differences in the expression of DMET genes have been documented. Excluding the effects of menstrual cycle, pregnancy, and application of contraceptives, Tanaka observed higher CYP3A4 activity in women than in men, in contrast to higher activities of CYP2C16, CYP2D6 and CYP2E1 in men than in women [6]. Reviewing others’ work, Scandlyn et al. concluded that CYP3A4 appeared to have a higher activity in women while CYP1A2 and CYP2E1 have higher activities in men [7]. By summarizing enzymatic activities from nearly 150 samples of human liver microsomes and 64 samples of human hepatocytes, Parkinson et al. concluded that there was no statistically significant difference in CYP3A4 activity between men and women in liver microsomes, but women had a two-fold higher CYP3A4 activity in their primary hepatocytes compared to men [8].

Sex differences in the expression of human DMET genes have been widely studied; however, most of the previous studies have been limited by sample size and/or the number of genes profiled. In addition, the common mechanisms involved in sexually differential regulation of DMETs in healthy human liver and their potential impact on drug therapy and public health are far from clear.

In the current study, previously published gene expression data derived from 234 male and 193 female human liver samples [9] was used to systemically analyze sex differences in the expression of 374 DMET genes in human liver. Co-expression networks were constructed to delineate the regulatory mechanisms involved in sex differences in the expression of human DMETs. Finally, the relationships between sexually dimorphic DMET genes and compounds regarding to clinical outcomes, molecular and cellular functions, and their implications to human diseases are discussed.

## Methods

### Gene expression data set

The dataset used for this analysis was from a previously published study [9,10] consisting of 427 liver samples consisting of 234 male and 193 female samples retrieved from three independent liver collections. The gene expression data were generated using an Agilent microarray platform with 39,302 probes corresponding to 19,541 genes. The microarrays were processed in a two-color mode using a common reference design. The expression level of a gene was expressed in the form of log10 ratio of its intensity value in the subject sample channel divided by the intensity value in the common reference channel.

### Identification of differentially expressed genes

To identify genes differentially expressed between the sexes, a fold change was calculated to represent the magnitude of the difference and a Student’s *t*-test was performed to estimate the statistical significance of the difference between 234 male and 193 female samples for each gene. Genes with a *P*>0.05 were eliminated, and the remaining genes were ranked by their absolute fold changes. A fold change cutoff value was applied to this ranked list of genes to determine which genes were differentially expressed. To identify the most differentially expressed genes from the entire set of genes profiled on the microarray, a fold-change (FC) cutoff of >1.5 was used in order to focus on a relatively small number of genes. For the identification of sexually dimorphic expression of DMET genes, a relatively small cutoff FC>1.1 was used in order to be able to examine as many differentially expressed genes as possible from the 374 DMET genes profiled on the microarray.

### Functional analysis of differentially expressed DMET genes

The identification of gene enrichment categories was determined according to the Gene Ontology (GO) categorization (http://www.geneontology.org/), Kyoto Encyclopedia of Genes and Genomes (KEGG) pathways (http://www.genome.jp/kegg/pathway.html), and SP-PIR keywords that combined the annotation from both Swiss-Prot (SP) and Protein Information Resource (PIR). Information on gene function, gene-chemical/drug interaction, and gene-disease relationship was obtained from GeneCards™ 3.0 (http://www.genecards.org/). The significance level was determined by Fisher’s exact test and Bonferroni correction for multiple category comparisons. The Novoseek score of the relevance of the chemical compound/drug to the gene was evaluated based on literature text-mining algorithms. The relationships between the top 10 sexually differentially expressed DMET genes and their corresponding top 5 related chemicals, as well as the top 5 related diseases were visualized using Cytoscape (http://www.cytoscape.org/).

### Construction of gene co-expression network

Co-expression networks have been applied to explore the functional similarities among groups of genes. Within a network, genes associated with specific biological processes usually are co-expressed and clustered which allows one to look at the overall gene-gene correlation structure at a high-throughput level [11]. The 3,548 sexually differentially expressed genes (corresponding to 3,835 probes), with a FC value greater than 1.1 in the expression levels between sexes were selected for constructing the gene co-expression networks [12]. A 3,548 by 3,548 matrix of the pair-wise Pearson correlation coefficients was constructed to represent the similarity between any two genes in terms of their expression profiles across the 427 liver samples. This sirted to an adjacency matrix by the function, *a_ij_* = |*cor*(*x_i_*, *x_j_*)|^*β*^ where a_ij_ denotes the connection strength between gene expressions x_i_ and x_j_ across 3,548 genes. The parameter β in the co-expression network is approximately scale-free [13]. The model fitting index R^2^ of the linear model that regresses log [p (k)] on log (k) was introduced to measure the fitting of the network to this scale-free topology, where k is the connectivity and p (k) is the probability density of the connectivity. A β value of 6 was chosen because it achieved a fitting index greater than 0.8. The adjacency matrix was further transformed into a topological overlap matrix (TOM) [14], in which the topological overlap between two genes reflects not only their direct interactions but also their indirect interactions through all the other genes in the network. The average linkage hierarchical clustering was applied to group genes based on the TOM. Genes within a module are of higher topological overlap with each other than with genes outside this module.

## Results

### General status of sex differences in human hepatic gene expression

We used a combination of P-value<0.05 and fold-change (FC)>1.5 to identify the set of genes that are most differentially expressed between male and female liver samples, resulting in a list of 80 genes from the entire list of 19,541 genes probed on the microarray. 19 of these genes were located on sex specific chromosomes, of which, 7 were on the X chromosome and 12 were on the Y chromosome. The 5 most differentially expressed genes were located on the sex chromosomes and showed more than 20-fold (FC>20) differences in signal intensity between male and female samples. The remaining 61 genes were found on autosomal chromosomes. Among these 80 genes, the expression levels of some genes were dominated by female samples while others were dominated by male samples. Ten DMET genes were found to be differentially expressed with more than 1.5-fold differences, including SLC3A1, CYP7A1, ACSL4, CYP3A7, GSTA1, CYP3A4, GSTA2, UGT2B17, SLC13A1 and ADH1A (first 10 genes in Table 1).

### Sexually differential expression of human DMET genes

To explore sex differences in the expression of human hepatic DMET genes, we focused on analysis of 374 DMET genes profiled on the microarray. With a relaxed FC cutoff value of 1.1 in addition to a P-value less than 0.05, 77 DMET genes were found to be sexually dimorphic in human hepatic expression (Table 1). The top 10 most differentially expressed DMET genes (ranked by FC values) based on sex were further analyzed using GeneCards™ (http://www.genecards.org/). Among these 10 genes, CYP7A1, CYP3A7, CYP3A4, and ADH1A are involved in phase I metabolism; ACSL4, GSTA1, GSTA2, and UGT2B17 are phase II metabolizing enzymes, while SLC3A1 and SLC13A1 are transporters. Table 2 lists the top 10 genes, the biological pathways and associated diseases represented as well as drugs/chemicals metabolized by these genes.

### Gene co-expression network analysis

The 3,548 sexually differentially expressed genes with a FC>1.1 and *P*<0.05 were selected for network construction. TOM analysis [14] was performed to examine modules consisting of highly interconnected expression traits within the co-expression network. The topological overlap between two genes reflects not only their direct interaction but also their indirect interactions through other genes in the network.

**Figure 1:**
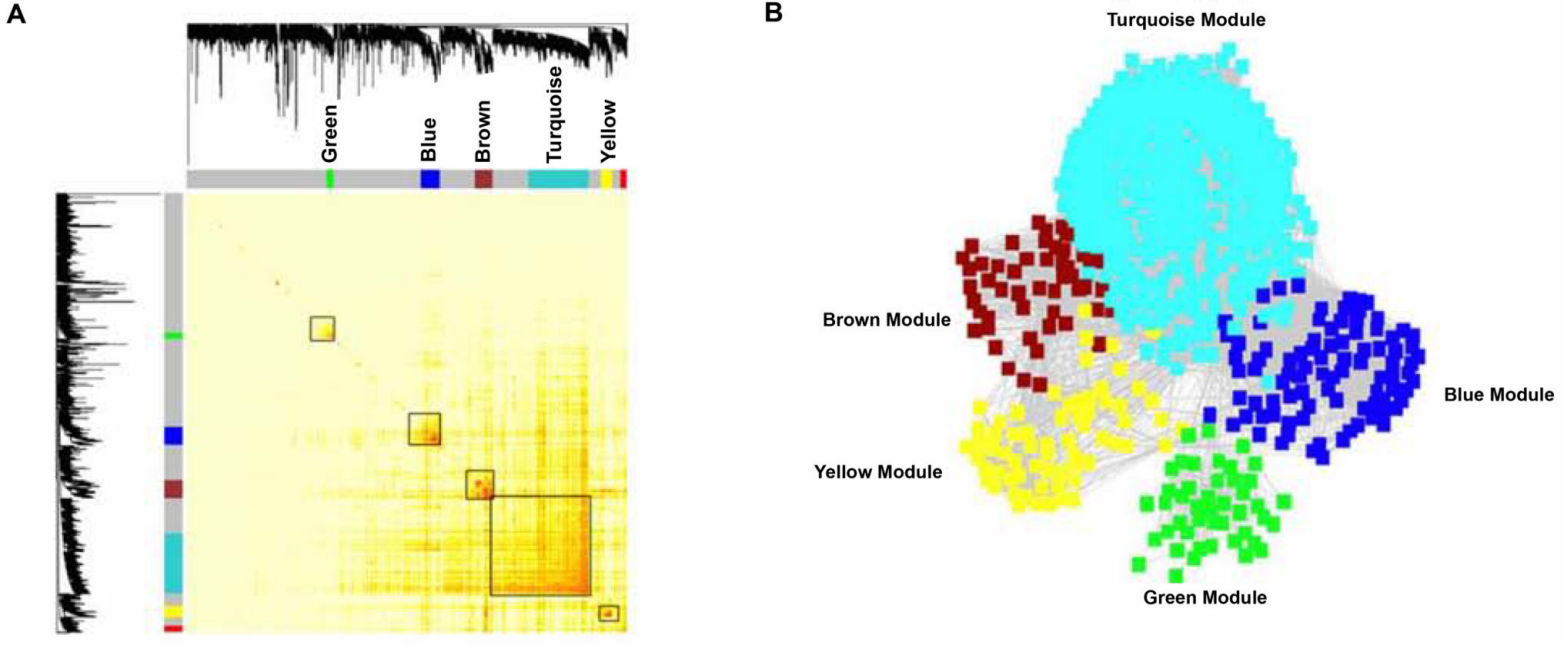
The Human Liver Gene Co-Expression Network of All Genes with Sex Differences (A) Topological overlap matrix (TOM) of all 3,548 sexually differentially expressed genes. Both the rows and the columns are sorted by hierarchical clustering. The colors specify the strength of the pair-wise topological connections (yellow: not significantly connected; orange: highly connected). Genes that are highly connected within a cluster are defined as a module. Each module was assigned a unique color identifier (turquoise, blue, green, yellow and brown), with the remaining genes colored gray; (B) The visualization of the co-expression network for sexually differentially expressed genes. The graph highlights that genes in the liver co-expression network fell into five distinct modules, where genes within a module were expressed with a higher correlation with each other than that of genes outside this module.

As illustrated by the TOM analysis (Figure 1A), five distinct modules were identified. Among the 3,548 sex-biased genes, 304 genes fell into these five modules, while the remaining 3,244 genes did not fall into any module. Since genes within a module are usually co-expressed together with a higher correlation than genes outside of the module, it can be inferred that genes within the same module have similarities in function or regulatory roles. To further infer the biological relevancy of genes within a module, gene enrichment analysis was performed for each module using the following functional databases: GO category, KEGG pathways, and SP-PIR keywords. Figure 1B highlights genes showing sexually dimorphic expression within each module and among different modules, indicating that these modules in the co-expression network were organized into different functional units. Biological functions listed in Table 3 showed that the five modules were significantly enriched by functional traits. The turquoise module, the largest module positively correlating with sex-based differential expression, was enriched with genes involved in oxidation/reduction, electron carrier, drug metabolism and fatty acid metabolism. This suggests that genes shown in the turquoise module are highly related to xenobiotic metabolism and transportation, since oxidation and reduction reactions are involved in major phase I drug-metabolism while electron transfer is associated with many phase III transport processes.

### Regulation network for sexually differentially expressed DMET genes

**Figure 2:**
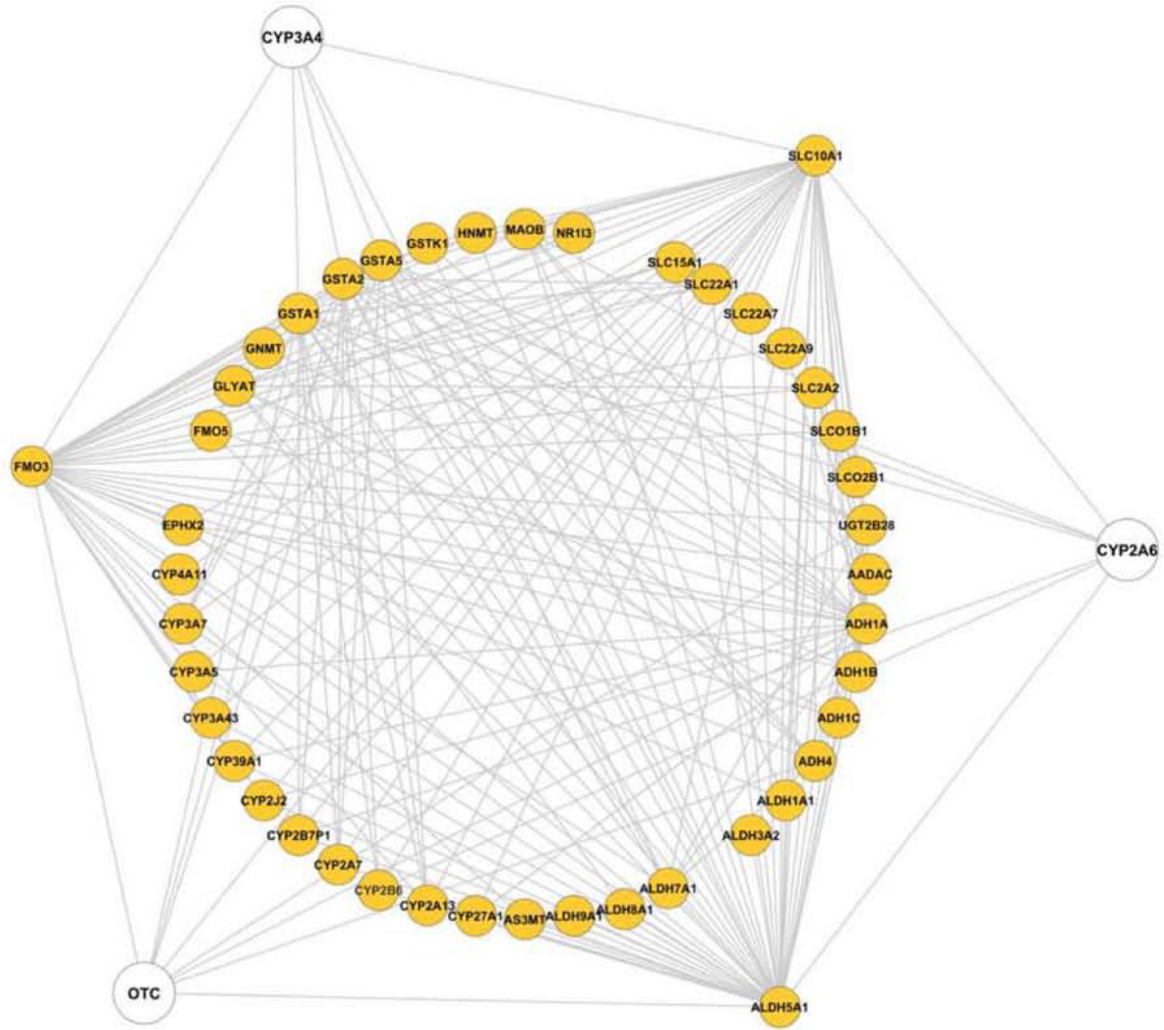
DMET Genes Co-Expression Network All sexually differentially expressed DMET genes are arranged in the inner circle. Three hub genes (FMO3, ALDH5A1 and SLC10A1), which have many more neighbors than others, are selected for a better visualization effect. Three genes CYP3A4, OTC and CYP2A6 with known expression regulatory mechanisms are in white.

Although sex differences in the expression of human DMET genes have been observed, the underlying biological mechanisms for such regulation are far from being fully understood. To search for common ground of such regulatory mechanisms, we constructed a co-expression network based on the expression of the sexually dimorphic DMET genes. In the network (Figure 2), a line between two genes indicates a similarity in the expression level of these genes across 427 liver samples, and thus may suggest commonality in the regulation of their expression. Figure 2 represents a global view of the network, displaying the co-expression relationship of these DMET genes that may imply putative regulatory pathways.

Growth hormone periodicity [15], sex hormonal control [16], and genetic differences [17] between the sexes are believed to be fundamental factors in regulating sexually dimorphic expression of genes. Dhir et al. [18] reported that CYP3A4 expression was increased by continuous treatment with growth hormone (masculine) and was suppressed by pulsatile treatment of growth hormone (feminine). In the co-expression network analysis, FMO3, GSTA1, GSTA2, GSTA5, ALDH5A1 and SLC10A1 showed similarities with CYP3A4, suggesting that the sexually dimorphic expression of these enzymes may have a mechanistic commonality with CYP3A4. The expression of CYP2A6 in humans can be induced by estrogen via its receptor [19], thus CYP2A6-connected genes, including ALDH5A1, CYP2B6, CYP2B7P1, SLC10A1, GSTA1, GSTA2, and GSTA5 in the network may share similar mechanisms for differential expression. Another major source of sex-biased gene expression is the difference between the inactive and active X chromosome genes regulated by both genetic (such as XIST gene products for the specific silencing of X-chromosome genes [20]) and epigenetic (such as altered histone acetylation and DNA methylation for gene silencing [21]) mechanisms. Although ornithine carbamoyltransferase (OTC) does not belong to DMETs, as an X-chromosome specific gene, regulation of its expression by the above mechanisms may provide insight for better understanding why some of the DMETs, such as GSTA1, GSTA2, GSTA5, SLC22A1, UGT2B28, ADH1A, ADH4, and ALDH5A1, show sexually dimorphic gene expression patterns. Interestingly, CYP3A4, CYP2A6 and OTC are all connected to GSTA1, GSTA2, GSTA5 and ALDH5A1, indicating that these genes may be involved in the crosstalk among sex hormone control, growth hormone control, and X chromosome specific gene clusters. Notably, with multiple connections with other DMETs, FMO3, SLC10A1 and ALDH5A1 also behaved as “hubs” in the network, indicating that they have expression similarities with other DMETs and thus may have more complicated mechanisms accounting for their sexually dimorphic expression.

### Role of DMET genes in human diseases and drug metabolism

DMET genes play important roles in human physiology and drug metabolism. The implication of differentially expressed DMET genes in drug metabolism and disease susceptibilities in a sex-dependent manner is of much interest. The interaction between differentially expressed DMETs and their metabolized endogenous and exogenous compounds (e.g., steroid hormones and drugs) and related susceptibilities to diseases (e.g., metabolic disorders and cancer), was analyzed by Novoseek analysis in GeneCards™. To display these associations, Cytoscape was used to integrate and visualize gene-chemical relationships and gene-disease relationships.

**Figure 3:**
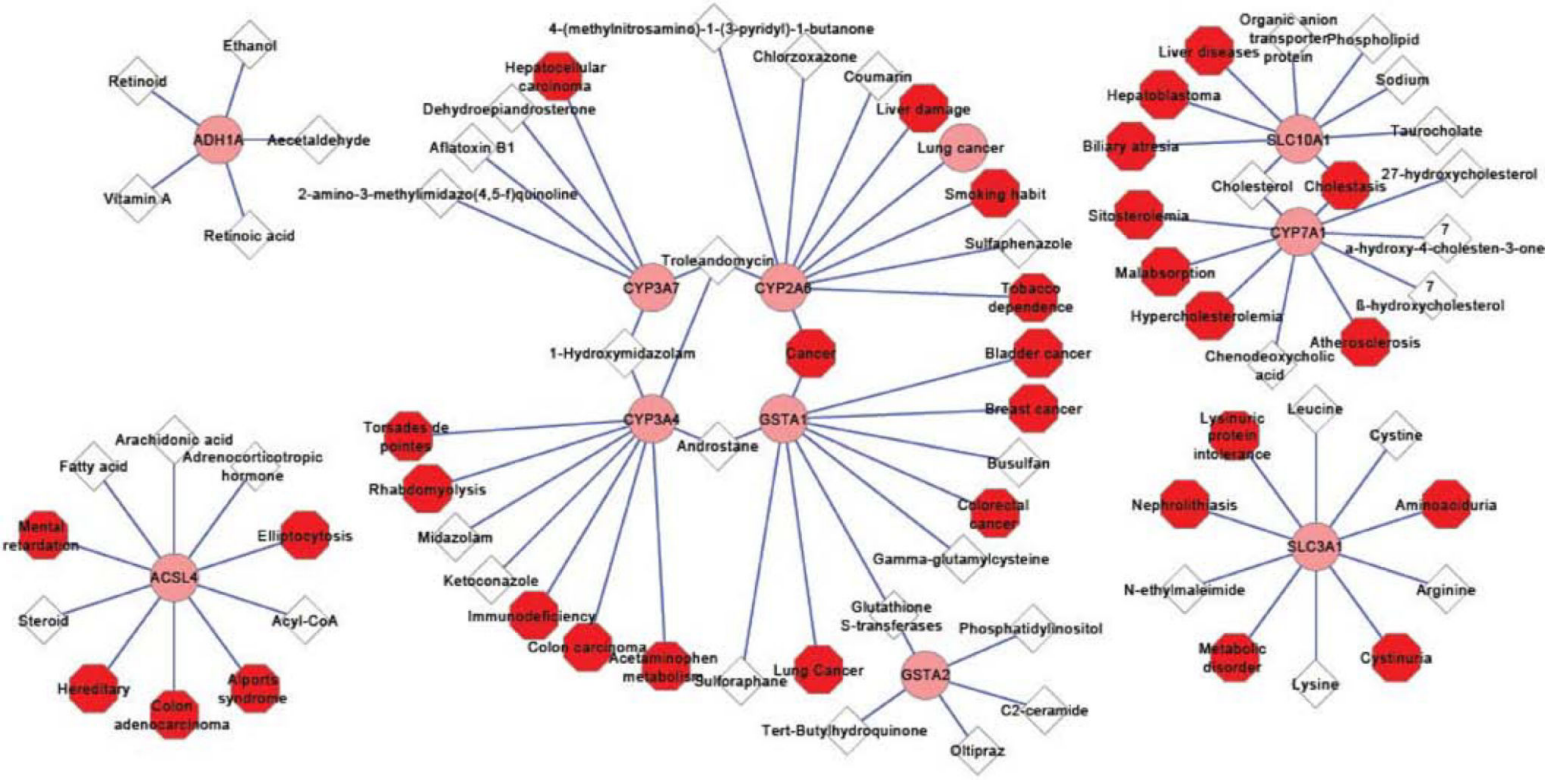
Interaction of DMET Genes with Compounds and Human Diseases Cytoscape was applied to depict the relationship between DMET genes and compounds and human diseases. Only the top 5 chemicals and the top 5 diseases associated with the top 10 sex-biased DMET genes were analyzed; and more details can be found in Supplementary Table 1. The following symbols and colors are used: pink circles for genes, white diamond for chemicals, and red octagons for human diseases.

The relationships between the top 10 sexually dimorphic DMET genes and related endogenous and exogenous compounds, as well as related human diseases were analyzed. Since many compounds and diseases may be related to a gene, only the top 5 ranked compounds and top 5 ranked diseases based on Novoseek scores are presented in Figure 3, and more detailed information for the contexts of such interactions are listed in the Supplement Table 1. As shown in Figure 3, the top 10 sexually dimorphic DMET genes have interactions with the metabolism of exogenous compounds and/or human diseases, and several of these genes share a similar relationship with the same group of compounds or are related to similar diseases. For example, hydroxylation activities of CYP2A6 and CYP3A7 could be inhibited by troleandomycin [22], and midazolam is metabolized both by CYP3A4 and CYP3A7 [23]. CYP2A6 and GSTA1 are both involved in metabolic activation of several procarcinogens, and thus have been linked (in expression levels or genotypes) to the etiology of cancers such as tobacco-related lung cancer [24], colorectal cancer [25] and breast cancer [26].

Interestingly, co-interaction of SLC10A1 and CYP7A1 with cholesterol is also depicted in Figure 3. Cholesterol homeostasis is balanced between dietary cholesterol uptake and endogenous cholesterol synthesis and excretion of bile acids. Bile acid synthesis from cholesterol is mediated by CYP7A1, an initial and classic alternative pathway, whereas SLC10A1 assists the hepatic uptake of bile acids as a sinusoidal Na^+^-bile acid co-transporter [27]. In children with early- and late-stage cholestasis, SLC10A1 and CYP7A1 were significantly downregulated [28], suggesting that these two genes contribute to cholestasis in human.

## Discussion

A major molecular factor involved in sex-related differences of drug responses and disease development is related to drug-metabolizing enzymes and drug transporters, and likely related to differential expression of DMET genes. However, very few studies have been done systematically to analyze the expression traits of a large panel of DMET genes in human liver with a sufficient sample size to reliably assess the nature of sexually differential expression of DMET genes. In this study, data were retrieved from a large cohort consisting of 427 human liver samples [10] to analyze the expression profile of 374 DMETs. This panel of DMETs included the majority of DMETs, and the size of the human liver sample cohort appears to be the largest to appear in the public domain.

Different gene expression patterns between males and females were observed for GSTs, SULTs, UGTs, and ATP-binding cassette (ABC) transporters [29]. Consistent with previous work, a large number of differentially expressed CYP 450s were observed, including several that have been previously reported such as CYP7A1 [30], CYP3A7, CYP3A4, CYP3A43 [31–33], CYP2A6 [34], CYP1B1 [35], CYP2A13 [36], and CYP2B6 [37]. In addition, other DMET genes displayed variable expression differences between genders including phase II metabolism enzymes such as GSTA1, GSTA2, SULT1C2 and UGT2B17, transporter SLC family members such as SLC3A1 and SLC10A1, and ABC family members such as ABCA12 and ABCA1. However, results were also observed that were not consistent with previous literature reports. For example, in this study, expression of ADH1 was 1.53 fold higher in females than in males, which differs from a previous report in which ADH1 expression was significantly higher in males than in females [38]. It is possible that different conclusions were drawn due to the limited sample size, with only 30 males and 20 females in the earlier report; however, other potential differences between the datasets could not be ruled out, such as dietary and medication influences on expression of DMETs.

Awareness of sex differences in response to drugs is clinically important. There is considerable evidence for gender-based differences in clinical studies. For example, CYP3A4-substrate drugs such as cyclosporine, erythromycin, tirilazad, verapamil, nifedipine, diazepam and alfentanil, have a higher clearance in women, which persists even after adjustments for physiological factors (e.g., body weight) [39]. Using 38 datasets containing clearance rates for 18 CYP3A substrate drugs measured in healthy men and women, it has been reported that the overall mean value for the female/male ratio of weight-normalized clearance was 1.26 for parenteral dosage and 1.17 for oral dosage. This result suggests that the sex difference in pharmacokinetics of CYP3A substrate drugs is clinically significant [40]. To determine gender differences in the efficacy and safety of commonly prescribed drugs, Gartlehner et al. analyzed data from 59 studies involving 250,000 patients and concluded that women had substantially lower response rates to antiemetics than men, men had higher rates of sexual dysfunction than women when treated with paroxetine for depression, and women experienced lovastatin-induced adverse events more frequently than men [41]. We believe that interindividual differences in drug metabolism are largely related to the expression of DMET genes, while the high expression/activity of hepatic CYP3A4 in women might partially account for the higher clearance for these drugs. The overall gender-based pharmacologic effects may not be caused by typically monogenetic traits (such as the expression level of CYP3A4); rather, they might be determined by interactions of several drug metabolizing enzymes and transporters involved in multiple pathways of drug metabolism, disposition, and drug targeting. For example, low dose administration of aspirin decreases the risk of stroke for women and the risk of myocardial infarction for men. Side effects of aspirin, gastrointestinal bleeding and peptic ulcer are reported to be significantly more common among women than men [42].

Sex differences in adverse drug reactions (ADRs) have drawn significant attention in recent years. Being female is known to be a risk factor for developing ADRs with data suggesting that women have a 1.5- to 1.7-fold greater risk of suffering ADRs than men [43]. A review by the U.S. General Accounting Office also showed that eight of the ten drugs withdrawn from the market during the period January 1, 1997 through December 2000 were due to greater risks of ADRs in women [44]. One aspect that can affect perceived sex bias is the number of women vs. men taking each drug. This report noted that 4 of the 8 drugs that were removed may have shown such a bias because these were prescribed more often to women than men. The other 4 drugs, however, did not exhibit this differential prescription rate.

Genetic make-up makes a huge difference in the gene expression between men and women, which in turn introduces gender-based differences in drug absorption, distribution, metabolism and excretion. If a drug is either not transformed at the anticipated rate (modulated by drug-metabolizing enzymes) or not effluxed/absorbed at the anticipated rate (modulated by transporters), elevated and/or prolonged exposure may occur. When the drug has a narrow therapeutic window relative to safety margin, such a pharmacokinetic difference could precipitate ADRs [45]. Although few studies in the literature could demonstrate that sexually dimorphic DMET gene expression is associated with different disease risks between genders, studies directly or indirectly showed that altered expression levels of DMET genes might change the incidences of various diseases. For example, expression differences in DMET genes such as CYP3A4, CYP2A6 and GSTA1 may be associated with cancer risks. CYP2A6 appears to activate several procarcinogens such as hexamethylphosphoramide, 4-(methylnitrosamino)-1-(3-pyridyl)-1-butanone (NNK) and aflatoxin B_1_, and studies have shown that the CYP2A6 activity is associated with pancreatic cancer [46] and colorectal cancer [25]. CYP3A4⋆1B conferred an increased risk for the development of prostate cancer through mediation of prostate cell growth and differentiation [47], while a functional study demonstrated that CYP3A4^⋆^1B enhances CYP3A4 expression by altering its promoter binding affinity to transcriptional factors compared to CYP3A4⋆1A [48]. GSTA1⋆1B, a polymorphism located in the promoter of GSTA1, is associated with decreased hepatic expression of GSTA1, which was discovered in a population study using human liver samples [49]. An epidemiological study demonstrated that decreased expression of GSTA1 is associated with an increased risk of colorectal cancer, especially in consumers of well-done red meat, since GSTA1 is involved in the detoxification pathway of food-born heterocyclic amines [50].

Generally, several known contributors have been reported to regulate the expression of DMETs, such as genetic components [51], epigenetic mechanisms [52], orphan nuclear receptors [53], and sex-hormone and/or growth-hormone regulated transcription factors [15]. Among these postulated mechanisms, sex hormones and growth hormones are thought to be the most important factors regulating sexually dimorphic expression of DMET genes. For example, there is evidence that many isoform-specific changes in DMET activities are mediated via sex hormones and/or growth hormones [54]. However, more studies are warranted to examine the underlying mechanisms responsible for hormonal-induced changes in sexually dimorphic DMET expression/activity. The co-expression network analysis in this study displayed commonalities of expression characteristics among sexually differentially expressed DMET genes, suggesting that bioinformatic approaches might be useful tools to identify underlying regulatory mechanisms for genes with similar expression patterns. Together with previous knowledge of possible pathways regulating the DMET gene expression in human liver, the gene-gene regulation network should help to better understand the global regulation mechanisms of sexually dimorphic expression of DMET genes.

Often information on age, medication history, chemical exposure, and disease status of donors of liver samples are unknown, confounding the results from *in vitro* studies of DMET expression and activity in human liver microsomal samples. These confounding factors also constrained the interpretation of results in the current study. However, taking the large sample size, a broad spectrum of DMETs and the systematic approach to analyze sexually dimorphic gene expression and its clinical implications into consideration, the present study should help to understand interindividual differences in drug/xenobiotics responses and human disease susceptibilities between males and females.

## Supplementary Material

Yang-DMT Supplemental

## Figures and Tables

**Table 1:** DMET genes with sex differences in human hepatic expression.

Number	Gene Symbol	Gene Title	P-value	Fold Change	Sex Biased
1	SLC3A1	solute carrier family 3 (cystine, dibasic and neutral amino acid transporters, activator of cystine, dibasic and neutral amino acid transport), member 1	7.27E-12	2.35	F
2	CYP7A1	cytochrome P450, family 7, subfamily A, polypeptide 1	1.28E-10	2.1	F
3	ACSL4	acyl-CoA synthetase long-chain family member 4	0.00266	2	F
4	CYP3A7	cytochrome P450, family 3, subfamily A, polypeptide 7	9.35E-08	1.83	F
5	GSTA1	glutathione S-transferase A1	0.000132	1.82	F
6	CYP3A4	cytochrome P450, family 3, subfamily A, polypeptide 4	5.25E-06	1.73	F
7	GSTA2	glutathione S-transferase A2	0.00266	1.69	F
8	UGT2B17	UDP glucuronosyltransferase 2 family, polypeptide B17	0.0002	1.59	M
9	SLC13A1	solute carrier family 13 (sodium/sulfate symporters), member 1	0.0166	1.57	M
10	ADH1A	alcohol dehydrogenase 1A (class I), alpha polypeptide	0.00003	1.53	F
11	CYP2A6	cytochrome P450, family 2, subfamily A, polypeptide 6	0.0147	1.49	F
12	SLC10A1	solute carrier family 10 (sodium/bile acid cotransporter family), member 1	0.00288	1.48	F
13	CYP2A7	cytochrome P450, family 2, subfamily A, polypeptide 7	0.0212	1.46	F
14	GSTA5	glutathione S-transferase A5	0.00496	1.43	F
15	CYP2A13	cytochrome P450, family 2, subfamily A, polypeptide 13	0.0272	1.43	F
16	HMGCR	3-hydroxy-3-methylglutaryl-Coenzyme A reductase	1.03E-06	1.39	F
17	GLYAT	glycine-N-acyltransferase	0.00223	1.38	F
18	SLC16A8	solute carrier family 16, member 8 (monocarboxylic acid transporter 3)	0.0419	1.35	F
19	FMO3	flavin containing monooxygenase 3	0.0025	1.34	F
20	ADH1C	alcohol dehydrogenase 1C (class I), gamma polypeptide	0.00801	1.34	M
21	CYP2B6	cytochrome P450, family 2, subfamily B, polypeptide 6	0.0265	1.33	F
22	ADH4	alcohol dehydrogenase 4 (class II), pi polypeptide	0.0367	1.33	F
23	CYP2B7P1	cytochrome P450, family 2, subfamily B, polypeptide 7 pseudogene 1	0.034	1.32	F
24	ADH1B	alcohol dehydrogenase 1B (class I), beta polypeptide	0.0116	1.31	F
25	EPHX2	epoxide hydrolase 2, cytoplasmic	0.00129	1.3	F
26	CYP3A43	cytochrome P450, family 3, subfamily A, polypeptide 43	0.00074	1.3	F
27	SLCO1B1	solute carrier organic anion transporter family, member 1B1	0.0151	1.29	F
28	CYP39A1	cytochrome P450, family 39, subfamily A, polypeptide 1	0.00138	1.29	F
29	ABCA12	ATP-binding cassette, sub-family A (ABC1), member 12	0.0133	1.29	M
30	SLC5A6	solute carrier family 5 (sodium-dependent vitamin transporter), member 6	8.32E-06	1.29	M
31	SLC16A14	solute carrier family 16, member 14 (monocarboxylic acid transporter 14)	0.0298	1.28	M
32	FMO1	flavin containing monooxygenase 1	4.37E-08	1.27	F
33	ALDH1B1	aldehyde dehydrogenase 1 family, member B1	0.0151	1.27	F
34	CYP3A5	cytochrome P450, family 3, subfamily A, polypeptide 5	0.00455	1.27	F
35	NR1I2	nuclear receptor subfamily 1, group I, member 2	0.00354	1.25	F
36	GNMT	glycine N-methyltransferase	0.0424	1.25	F
37	UGT2B28	UDP glucuronosyltransferase 2 family, polypeptide B28	0.0344	1.25	F
38	UGT2A3	UDP glucuronosyltransferase 2 family, polypeptide A3	0.00407	1.24	F
39	SLC22A7	solute carrier family 22 (organic anion transporter), member 7	0.0103	1.24	F
40	ALDH1A1	aldehyde dehydrogenase 1 family, member A1	0.00812	1.23	F
41	SLC22A1	solute carrier family 22 (organic cation transporter), member 1	0.0125	1.22	F
42	AADAC	arylacetamide deacetylase (esterase)	0.00517	1.22	F
43	BAAT	bile acid Coenzyme A: amino acid N-acyltransferase (glycine N-choloyltransferase)	0.0242	1.22	F
44	CES4	carboxylesterase 4 (monocyte/macrophage serine esterase 4)	0.0158	1.22	F
45	SLCO4A1	solute carrier organic anion transporter family, member 4A1	0.000551	1.22	M
46	ADH7	alcohol dehydrogenase 7 (class IV), mu or sigma polypeptide	0.0252	1.21	F
47	ALDH7A1	aldehyde dehydrogenase 7 family, member A1	0.00186	1.21	F
48	NNMT	nicotinamide N-methyltransferase	0.000628	1.21	M
49	UGT2B10	UDP glucuronosyltransferase 2 family, polypeptide B10	0.0345	1.2	F
50	CBR1	carbonyl reductase 1	0.000228	1.2	F
51	ALDH5A1	aldehyde dehydrogenase 5 family, member A1 (succinate-semialdehyde dehydrogenase)	0.00441	1.2	F
52	CYP51A1	cytochrome P450, family 51, subfamily A, polypeptide 1	0.000344	1.2	F
53	GPX2	glutathione peroxidase 2 (gastrointestinal)	0.000185	1.2	M
54	ORM2	orosomucoid 2	0.0023	1.2	M
55	HNMT	histamine N-methyltransferase	0.00187	1.19	F
56	FMO5	flavin containing monooxygenase 5	0.034	1.19	F
57	MAOB	monoamine oxidase B	0.0106	1.19	F
58	CYP2J2	cytochrome P450, family 2, subfamily J, polypeptide 2	0.00155	1.19	F
59	ORM1	orosomucoid 1	0.00164	1.19	M
60	CHST9	carbohydrate (N-acetylgalactosamine 4-0) sulfotransferase 9	0.00038	1.18	F
61	SLC2A2	solute carrier family 2 (facilitated glucose transporter), member 2	0.0355	1.18	F
62	SLC19A2	solute carrier family 19 (thiamine transporter), member 2	0.0116	1.18	F
63	ABCA2	ATP-binding cassette, sub-family A (ABC1), member 2	4.61E-06	1.17	F
64	SAT1	spermidine/spermine N1-acetyltransferase 1	0.00121	1.17	F
65	SLC16A9	solute carrier family 16, member 9 (monocarboxylic acid transporter 9)	0.0261	1.17	F
66	SLC10A2	solute carrier family 10 (sodium/bile acid cotransporter family), member 2	0.00207	1.17	M
67	ABCA1	ATP-binding cassette, sub-family A (ABC1), member 1	0.000414	1.17	M
68	ACSL1	acyl-CoA synthetase long-chain family member 1	0.0122	1.16	F
69	CYP27A1	cytochrome P450, family 27, subfamily A, polypeptide 1	0.00892	1.16	F
70	CYP4Z1	cytochrome P450, family 4, subfamily Z, polypeptide 1	0.0123	1.16	F
71	GPX3	glutathione peroxidase 3 (plasma)	0.0000818	1.16	M
72	CES1	carboxylesterase 1 (monocyte/macrophage serine esterase 1)	0.018	1.15	F
73	SULT1C2	sulfotransferase family, cytosolic, 1C, member 2	0.0323	1.15	M
74	SLC22A4	solute carrier family 22 (organic cation transporter), member 4	0.00243	1.14	M
75	ABCB1	ATP-binding cassette, sub-family B (MDR/TAP), member 1	4.72E-06	1.13	M
76	SLC22A23	solute carrier family 22, member 23	0.0134	1.13	M
77	CYP1B1	cytochrome P450, family 1, subfamily B, polypeptide 1	0.0152	1.13	M

**Table 2:** Top 10 of the most sexually differentially expressed DMETs and their biological functions.

Gene Symbol	Sexually Dimorphic Changes (Fold Chang)	*P*-value	Top 5 of Related Drugs	Major Biological Functions/Pathways
**SLC3A1**	2.35	7.27×10^−12^	N/A	Carbohydrate/cellular amino acid metabolism, ion/amino acid/basic amino acid/Lysine/transmembrane transport
**CYP7A1**	2.1	1.28 × 10^−10^	N/A	Bile acid biosynthetic process, cholesterol catabolic process, xenobiotic/steroid/bile acid/cellular lipid metabolism, cholesterol homeostasis, oxidation-reduction process, regulation of bile acid biosynthetic process, cellular response to glucose stimulus/cholesterol
**ACSL4**	2	2.66×10^−3^	N/A	Lipid/fatty acid/triglyceride/cellular lipid metabolism, response to nutrient, learning or memory, fatty acid transport, dendrite development, triglyceride biosynthetic process, long-chain fatty-acyl-CoA biosynthetic process, embryonic process involved in female pregnancy/response to interleukin-15
**CYP3A7**	1.83	9.35×10^−8^	Cisapride, Idazolam, Vitamin D, Xenobiotics	Xenobiotic metabolic process, oxidation-reduction process
**GSTA1**	1.82	1.32×10^−4^	Busulfan, Chlorambucil, Cyclophosphamide, Doxorubicin, Etoposide	Glutathione/xenobiotic metabolism
**CYP3A4**	1.73	5.25×10^−6^	Alprazolam, Anthracycline, Asparaginase, Cisapride,Citalopram	Lipid/xenobiotic/steroid/androgen/monoterpenoid/drug/vitamin D/heterocycle metabolic process, steroid/alkaloid/exogenous drug catabolism, oxidation-reduction process, oxidative demethylation
**GSTA2**	1.69	2.66×10^−3^	N/A	Glutathione/xenobiotic metabolism
**UGT2B17**	1.59	2.00×10^−4^	Losartan	Metabolic/steroid metabolic process/retinoic acidbinding/glucuronosyltransferase activity/transferase activity/transferring hexosyl groups
**SLC13A1**	1.57	1.66×10^−2^	Succinic acid	Transporter activity/symporter activity/sodium:sulfate symporter activity/ion transport/dium ion transport/sulfate transport/transmembrane transport
**ADH1A**	1.53	3.00×10^−5^	N/A	Alcohol/xenobiotic metabolism, ethanol oxidation, oxidation-reduction process

**Table 3:** Top enrichment terms for the five modules.

Module	Category	Term	Count	%	*P*-value
Yellow	KEGG PATHWAY	Ribosome	14	56	4.34E-22
	GOTERM_CC_FAT	Ribosomal subunit	13	52	3.55E-21
Turquoise	SP_PIR_KEYWORDS	Oxidoreductase	62	26.61	9.16E-42
	GOTERM_BP_FAT	Oxidation reduction	64	27.47	4.39E-37
	GOTERM_MF_FAT	Electron carrier activity	29	12.45	1.74E-18
	KEGG_PATHWAY	Drug metabolism	19	8.15	7.67E-16
	KEGG_PATHWAY	Fatty acid metabolism	16	6.87	3.07E-15
Blue	GOTERM_BP_FAT	Wound healing	7	9.86	6.18E-05
	GOTERM_BP_FAT	Response to wounding	10	14.08	1.03E-04
	GOTERM_BP_FAT	Rlatelet activation	4	5.63	2.06E-04
Green	SP_PIR_KEYWORDS	Acetylation	13	43.33	6.34E-05
	GOTERM_BP_FAT	Translational elongation	4	13.33	5.61E-04
	GOTERM_CC_FAT	Cytosolic ribosome	3	10	6.32E-03
Brown	SP_PIR_KEYWORDS	Protein biosynthesis	13	27.08	6.26E-15
	GOTERM_CC_FAT	Cytosolic ribosome	11	22.92	3.22E-14
	GOTERM_BP_FAT	Translational elongation	11	22.92	5.36E-14
	KEGG_PATHWAY	Ribosome	11	22.92	3.84E-13

## Abbreviations

DMET: Drug Metabolizing Enzyme and Transport
